# Supplementary material for: Geographical variation in hotspots of stunting among under-five children in Ethiopia: A geographically weighted regression and multilevel robust Poisson regression analysis
Source: PLoS One. 2024 May 14;19(5):e0303071. doi: 10.1371/journal.pone.0303071 (PMC11093352; doi:10.1371/journal.pone.0303071)
Supplement: Attachment — Submitted filename: renamed_fac9b.docx [file pone.0303071.s001.docx]

**Comments to Author’s - PONE-D-23-24168**

In this study, our aim was to investigate spatial disparities in the prevalence of stunted growth among children under the age of five in Ethiopia. Additionally, we explored how socioeconomic factors vary across space and influence stunted growth in the same context. While the topic under consideration is of significant importance, our study lacks a comprehensive contextualization within the existing body of research, particularly with respect to similar studies conducted using comparable methods in the same country. It is imperative to elucidate the unique contributions our study brings to the field of research. Consequently, substantial enhancements are required in the major sections of the paper to align it with the standards necessary for publication. Please refer to the detailed comments provided below for specific improvements.

Introduction

1. **Providing Context and Rationale for the Study**: Considering the existing body of research in Ethiopia on stunted growth, which often employs hotspot analysis as a common methodological approach, it is crucial to establish the unique contributions of our study. Furthermore, the absence of citations to these pertinent studies in our paper raises questions about the comprehensiveness of our literature review, including those published within this journal. To rectify this, I recommend revisiting the introduction section to incorporate references to these studies and elucidate the rationale for our study. Please refer to the specific studies mentioned for guidance and justification.

Haile, D., Azage, M., Mola, T. et al. Exploring spatial variations and factors associated with childhood stunting in Ethiopia: spatial and multilevel analysis. BMC Pediatr 16, 49 (2016). <https://doi.org/10.1186/s12887-016-0587-9>

Kuse, K.A., Debeko, D.D. Spatial distribution and determinants of stunting, wasting and underweight in children under-five in Ethiopia. BMC Public Health 23, 641 (2023). <https://doi.org/10.1186/s12889-023-15488-z>

Hagos S, Hailemariam D, WoldeHanna T, Lindtjørn B (2017) Spatial heterogeneity and risk factors for stunting among children under age five in Ethiopia: A Bayesian geo-statistical model. PLoS ONE 12(2): e0170785. <https://doi.org/10.1371/journal.pone.0170785>

Muche A, Melaku MS, Amsalu ET, Adane M (2021) Using geographically weighted regression analysis to cluster under-nutrition and its predictors among under-five children in Ethiopia: Evidence from demographic and health survey. PLoS ONE 16(5): e0248156. https://doi.org/10.1371/journal.pone.0248156

2**. Justifying the Importance of Spatial Heterogeneity**: In the introduction, it is imperative to provide a compelling argument for the significance of investigating spatial heterogeneity in predictors of stunted growth. To bolster this justification, it is essential to draw upon insights from prior studies that have employed this method to explore stunted growth or malnutrition. Demonstrating how these findings have contributed to the field and explaining how they lend weight to our study's objectives will enhance the rationale for our research. For reference and context, please consider examining the studies mentioned.

Amegbor, P.M., Zhang, Z., Dalgaard, R. et al. Multilevel and spatial analyses of childhood malnutrition in Uganda: examining individual and contextual factors. Sci Rep 10, 20019 (2020). <https://doi.org/10.1038/s41598-020-76856-y>

Muche A, Melaku MS, Amsalu ET, Adane M (2021) Using geographically weighted regression analysis to cluster under-nutrition and its predictors among under-five children in Ethiopia: Evidence from demographic and health survey. PLoS ONE 16(5): e0248156. https://doi.org/10.1371/journal.pone.0248156

Biswas, M. Identifying Geographical Heterogeneity in Associations between Under-Five Child Nutritional Status and Its Correlates Across Indian Districts. Spat Demogr 10, 143–187 (2022). <https://doi.org/10.1007/s40980-022-00104-2>

3**. Page 4 line 79 to 81**: please provide a reference

Data and Methods

4. **Measures for Spatial Analysis**: It is essential for the authors to clearly define how stunted growth was measured at the Enumeration Area (EA) level in both the Ordinary Least Squares (OLS) and Geographically Weighted Regression (GWR) models. Specify whether stunted growth was expressed as a rate per EAs, an absolute count, or using another metric to eliminate ambiguity.

5. **Modified Poisson Regression**: The description of the use of modified Poisson regression in the manuscript should be revised for clarity. The current explanation is not easily comprehensible. I recommend consulting the provided reference for guidance on how to present the use of modified Poisson regression effectively.

Amegbor, P. M. (2022). Early-life environmental exposures and anaemia among children under age five in Sub-Saharan Africa: An insight from the Demographic & Health Surveys. Science of the Total Environment, 832, 154957.

6. **Data Management and Analysis**: The section related to data management and analysis should be revised to provide more detailed information, especially concerning the process of merging survey data with GPS data. It would be valuable to explain how this integration was conducted. You may refer to the mentioned references for insight on how to structure this section effectively.

7. **Use of GWR**: The authors should address the choice of Geographically Weighted Regression (GWR) over alternatives such as Multiscale Geographically Weighted Regression (MGWR) in light of the known limitations of GWR. Additionally, clarify whether fixed or adaptive bandwidth was employed and provide a rationale for this choice. Specify which search option was used for selecting the optimal bandwidth. To justify the choice, consider referring to the provided references for further insights.

Fotheringham, A. S., Yang, W. & Kang, W. Multiscale geographically weighted regression (MGWR). Ann. Am. Assoc. Geogr. 107, 1247–1265 (2017).

Amegbor, P.M., Zhang, Z., Dalgaard, R. et al. Multilevel and spatial analyses of childhood malnutrition in Uganda: examining individual and contextual factors. Sci Rep 10, 20019 (2020). https://doi.org/10.1038/s41598-020-76856-y

Also, provide the information on whether fixed or adaptive bandwidth was used and why? And which search option was used for selecting the optimal bandwidth.

Results

8**. Page 13, Lines 205-207**: It is recommended to rewrite the sentence for clarity. Specify which models are being compared, as the current wording is confusing. While the Mixed Effect Poisson model is mentioned, it's unclear which other model or models are being referred to.

9. **Table 3**: Regarding Table 3, the choice of categorizing mother age below 20 should be explained, especially considering there was a significant association for mother age above 40 in the Mixed Effect Poisson model. Additionally, clarify that the table represents the proportion of mothers in poor households, as suggested.

Discussion

10. **Grounding the Discussion**: In the discussion section, it's important to contextualize the study's findings within the broader Sub-Saharan Africa (SSA) region, rather than referring to China. Given the prevalence of studies on stunted growth in SSA, consider comparing your findings with regional prevalence and explaining potential factors accounting for similarities, differences, or disparities. You may refer to the provided references for guidance on contextualizing your findings in the SSA context.

Amegbor, P.M., Sabel, C.E., Mortensen, L.H. et al. Early-life air pollution and green space exposures as determinants of stunting among children under age five in Sub-Saharan Africa. J Expo Sci Environ Epidemiol (2023). <https://doi.org/10.1038/s41370-023-00572-8>

Quamme, S. H., & Iversen, P. O. (2022). Prevalence of child stunting in Sub-Saharan Africa and its risk factors. Clinical Nutrition Open Science, 42, 49-61.

Tusting, L. S., Bradley, J., Bhatt, S., Gibson, H. S., Weiss, D. J., Shenton, F. C., & Lindsay, S. W. (2020). Environmental temperature and growth faltering in African children: a cross-sectional study. The Lancet Planetary Health, 4(3), e116-e123.

11. **Regional Disparities**: Provide explanations for the high prevalence of stunted growth in specific regions, such as Tigray, central, northeast, and southern Amhara, north, west, and south Afar, and east SNNPR. Analyze local or regional factors that could account for these disparities to make the discussion more contextualized and specific.

12**. Interpreting GWR Results**: Avoid using the term "hotspots" when discussing GWR results, as the method does not conceptually measure hotspots. Instead, focus on explaining the spatial variation in associations between predictors and stunted growth in a more methodologically accurate manner.

13**. GWR Results**: Analyze the GWR results in-depth, particularly the positive associations of all predictors with stunted growth, except for poverty and male household heads. If maternal education and other factors had a global effect, consider exploring alternatives like Spatially Varying Coefficient Models (e.g., SGWR or Semi-Parametric GWR) in the absence of MGWR.

14. **Contextualize GWR Discussion**: Given that poverty and male household heads show both positive and negative associations in different areas, delve into why such spatial variations in association exist. Explore the reasons behind the positive association in some places and negative association in others, emphasizing the spatial variation in association, which is a central motivation for the study.
