# Supplementary material for: Geographical variation in hotspots of stunting among under-five children in Ethiopia: A geographically weighted regression and multilevel robust Poisson regression analysis
Source: PLoS One. 2024 May 14;19(5):e0303071. doi: 10.1371/journal.pone.0303071 (PMC11093352; doi:10.1371/journal.pone.0303071)
Supplement: Attachment — Submitted filename: Manuscript review results_200923.docx [file pone.0303071.s002.docx]

Dear Author,

Please consider the following observations and revise accordingly:

1. The author should correct this manuscript: In Ethiopia Stunting has steadily decreased, from 58% 71 in 2000 (2) to 36.81% in 2019 (3).
2. In the Introduction section: there is no explanation of the geographical conditions of stunting hotspots in Ethiopia.
3. What are the limitations of using DHS secondary data? And how to overcome these limitations?
4. How to calculate the sample size of 5490 children under five? What are the author's considerations in calculating the sample size?
5. The author corrected the punctuation in this manuscript: Dependent variable: In this study, the dependent variable was stunting, which was classified as "Yes = 1" for a child with a length or height/age -2 Z score and "No = 0" for a child with a height/age > -2 Z score.
6. Ethical considerations: How did the authors obtain ethical considerations for the use of secondary data? For example, for permissions and approval of data use.
7. On discussion: On discussion: what recommendations are given by the author in stunting prevention based on the findings of this study?
8. How does it compare with the results of previous studies?
