# Supplementary material for: Geographical variation in hotspots of stunting among under-five children in Ethiopia: A geographically weighted regression and multilevel robust Poisson regression analysis
Source: PLoS One. 2024 May 14;19(5):e0303071. doi: 10.1371/journal.pone.0303071 (PMC11093352; doi:10.1371/journal.pone.0303071)
Supplement: Attachment — Submitted filename: Point by point response.docx [file pone.0303071.s003.docx]

# **Point-by-point response**

**Point by point response for editors/reviewers comments**

PLOS ONE

**Manuscript title:** Geographical variation in hotspots of stunting among under-five children in Ethiopia using the 2019 Demographic and Health Survey: a geographically weighted regression and multilevel robust Poisson regression analysis

**Manuscript ID:**  PONE-D-23-24168

**Dear editor/reviewer.**

Dear all,

We would like to thank you for the constructive, building, and improvable comments on this manuscript that would improve the content of the manuscript. We considered each comment and clarification question of editors and reviewers on the manuscript thoroughly. Our point-by-point responses for each comment and question are described in detail on the following pages. Further, the details of changes were shown by track changes in the supplementary document attached

**Additional Editor Comments:**

The manuscript has been evaluated by two reviewers, and one reviewer has major concerns.
Could you please carefully revise the manuscript to address all comments raised. In particular, could you please pay special attention to the second comment raised by the reviewer, and expand on how you conducted the MGWR test?

**Author’s response: Dear Academic editor thank you for your concerns.** As mentioned by the reviewer, ArcGIS does not support MGWR. Therefore, we used the MGWR 2.2 statistical software to run the MGWR test for our dataset. Since GWR was the best-fitted model for our data, we ran the GWR model using ArcGIS software. We have included the results of the model diagnostic tests below.

**'Response to Reviewers**

Methods:
1. Sample Size: In accordance with Reviewer 2’s comments, the authors should provide information on how they arrived at the final sample size of 5,490 (Page 6, lines 126 to 127).

**Author’s response:** Given that we already utilized a nationally representative dataset with an appropriate sample size, recalculating the sample size seems unnecessary. As you know, DHS surveys are representative at the national level, for urban and rural areas. All survey-sampling strategies are subject to sampling error. The DHS Program designs samples to provide national and subnational estimates with a reasonable relative standard error. The larger the sample size, the smaller the relative standard error on any given indicator will be. Therefore, we do not calculate sample size while we use this data we used the available sample as it is. By utilizing the available sample as-is, we are leveraging the existing data efficiently. This approach ensures that the estimates are robust while minimizing unnecessary recalculations.

2. Comment 7 – Use of GWR: In response to my initial comment on the limitations of GWR and the advice for the authors to consider MGWR, the authors claim they used AUCc and R2 to determine the best-fitting model. To the best of my knowledge, the analytic platform used for spatial analysis, ARCGIS 10.7, doesn’t support MGWR testing or lacks MGWR functionality. Thus, how did the authors conduct the MGWR test? Additionally, the authors should provide the test diagnostics information for both the GWR and MGWR models in their response. Note that the developer of GWR strongly recommends the use of MGWR due to the known limitations of GWR; hence, I encourage the authors to read the Fotheringham et al. paper referenced in comment 7 in my previous assessment

**Author’s response:** Dear reviewer thank you for your suggestion. Given that ArcGIS does not support MGWR, we employed the MGWR 2.2 statistical software to conduct the MGWR test. Notably, we found the GWR model to be the best-fitted model for our dataset. Consequently, we utilized ArcGIS software to run the GWR model.

Here is the test diagnostic information for both models from the MGWR 2.2 software

| diagnostic information | GWR | MGWR |
| --- | --- | --- |
| AIC | 457.894 | 778.972 |
| AICc | 459.482 | 781.815 |
| BIC | 512.459 | 851.930 |
| Adjusted R-square | 0.37 | 0.29 |

Discussion:

3. Page 21, lines 323 to 324: Please clarify this sentence: “The prevalence of stunting 324 is consistent with a 33 (8) and 55 (30) SSA countries and East African study (31).” It is incomprehensible in its current form.

**Author’s response:** Dear reviewer thank you for the comment. We have rewrite the aforementioned sentence. (Please see the revised manuscript)

4. Comment 12 – Use of Hotspot for GWR Result Interpretation: Despite my previous caution against using the term "hotspot" for GWR results, it still appears in the revised manuscript (e.g., Page 23, lines 366). Please refrain from using "hotspot" in reference to GWR results. Refer to the suggested reference for the correct interpretation of the GWR results.

**Author’s response:** Dear reviewer thank you for the comment. It was an editorial mistake we have addressed it.

5. Discussion of Poverty and Stunted Growth GWR Results: The authors need to clarify whether the intervention programs cited as offsetting the effect of poverty in some areas are specifically for those communities and provide a reference to that effect. If they are nationwide programs, then the authors should explain why they affect some communities positively and have no effect in other communities.

**Author’s response:** Dear reviewer thank you for the comment. We have provided a reference for nutritional interventions and policy implementations that apply to specific regions. Additionally, we have included possible reasons for why these interventions have diverse outcomes in different regions. For future researchers, it would be extremely valuable to investigate the reasons behind the varying effects of these interventions in different regions.
