# Supplementary material for: Geographical variation in hotspots of stunting among under-five children in Ethiopia: A geographically weighted regression and multilevel robust Poisson regression analysis
Source: PLoS One. 2024 May 14;19(5):e0303071. doi: 10.1371/journal.pone.0303071 (PMC11093352; doi:10.1371/journal.pone.0303071)
Supplement: Attachment — Submitted filename: Comments to Authors.docx [file pone.0303071.s004.docx]

Comments to Authors

Thank you for addressing such interesting public health concern.

**Abstract**

Line 46…. rich household wealth index. I suggest to replace this phrase with “household wealth status”.

**Method and materials**

Line 141… community maternal illiteracy level… please use maternal literacy level instead of maternal illiteracy level.

Line 179: Did the authors check the assumption of spatial regression analysis?

**Results**

**Table 1.** The way by which authors compute household wealth index is not explained in methodology part. I suggest the authors explain how many variables were used to compute household wealth index? Did you check the assumption of PCA?...... need to be explained in the methodology section.

**Table: 1** Source of drinking water

Improved, not improved… What is improved and not source of drinking water? I suggest these terms need to be operationalized.

**Table :3**

Proportion of mothers with poverty…. please replace this phrase with “proportion of mother with poor household wealth status”

**Discussion**

The discussion is shallow, and need to be discussed by adding more previous studies across the world.

Line 338…Tigray has been affected by armed conflict, leading to displacement, disrupted healthcare services, and food insecurity... As to me this cannot be a justification because the war in Tigray region was erupted in November 2020. The authors used 2019 EMDHS data which is before the war. Thus, the consequence of Tigray armed conflict cannot justify the discrepancy.

-Significantly associated variables in the geographically weighted regression were not well discussed. I suggest the authors compare this finding by adding more previous studies across the countries.

**Conclusion**

**-**Please clearly add the implication of the finding.
