# Supplementary material for: Geographical variation in hotspots of stunting among under-five children in Ethiopia: A geographically weighted regression and multilevel robust Poisson regression analysis
Source: PLoS One. 2024 May 14;19(5):e0303071. doi: 10.1371/journal.pone.0303071 (PMC11093352; doi:10.1371/journal.pone.0303071)
Supplement: Attachment — Submitted filename: Point by point response.docx [file pone.0303071.s005.docx]

# **Point-by-point response**

**Point by point response for editors/reviewers comments**

PLOS ONE

**Manuscript title:** Geographical variation in hotspots of stunting among under-five children in Ethiopia using the 2019 Demographic and Health Survey: a geographically weighted regression and multilevel robust Poisson regression analysis

**Manuscript ID:**  PONE-D-23-24168

**Dear editor/reviewer.**

Dear all,

We would like to thank you for the constructive, building, and improvable comments on this manuscript that would improve the content of the manuscript. We considered each comment and clarification question of editors and reviewers on the manuscript thoroughly. Our point-by-point responses for each comment and question are described in detail on the following pages. Further, the details of changes were shown by track changes in the supplementary document attached

**'Response to Reviewers**

**Abstract**

Line 46…. rich household wealth index. I suggest to replace this phrase with “household wealth status”.

**Author’s response:** Dear reviewer thank you for your comment. We have replaced the phrase with “household wealth status”.

**Method and materials**

Line 141… community maternal illiteracy level… please use maternal literacy level instead of maternal illiteracy level.

**Author’s response:** Dear reviewer thank you for your comment. We have replaced the phrase with “maternal literacy level”.

Line 179: Did the authors check the assumption of spatial regression analysis?

**Author’s response:** Before proceeding to the local model, the six assumptions of the OLS model (the explanatory variables should have the expected relationship, the significance of each explanatory variable, the randomness of residuals, assuring the statistical no significance of Jarque-Bera statistics, Variance Inflation Factor (VIF) value, and the strength of R-square) were checked. The results for the tests of those assumptions were presented in the manuscript (Table 3 and 4).

**Results**

**Table 1.** The way by which authors compute household wealth index is not explained in methodology part. I suggest the authors explain how many variables were used to compute household wealth index? Did you check the assumption of PCA?...... need to be explained in the methodology section.

**Author’s response:** we do not compute household wealth index because in the Demographic and Health Surveys (DHS), the household wealth index is indeed constructed using household asset data via principal components analysis (PCA). This index provides a measure of relative wealth or socioeconomic status for each household surveyed.

<https://www.dhsprogram.com/topics/wealth-index/Wealth-Index-Construction.cfm>

**Table: 1** Source of drinking water

Improved, not improved… What is improved and not source of drinking water? I suggest these terms need to be operationalized.

**Author’s response:** Dear reviewer thank you for the suggestion. We have included the operational definition for source of drinking water.

**Table :3**

Proportion of mothers with poverty…. please replace this phrase with “proportion of mother with poor household wealth status”

**Author’s response:** Thank you dear reviewer for your comment. We have replaced the term with “proportion of mother with poor household wealth status”

**Discussion**

The discussion is shallow, and need to be discussed by adding more previous studies across the world.

Line 338…Tigray has been affected by armed conflict, leading to displacement, disrupted healthcare services, and food insecurity... As to me this cannot be a justification because the war in Tigray region was erupted in November 2020. The authors used 2019 EMDHS data which is before the war. Thus, the consequence of Tigray armed conflict cannot justify the discrepancy.

-Significantly associated variables in the geographically weighted regression were not well discussed. I suggest the authors compare this finding by adding more previous studies across the countries.

**Author’s response:** Thank you dear reviewer for your comment. We have thoroughly revised our discussion by adding a multitude of pertinent previous studies.

**Conclusion**

**-**Please clearly add the implication of the finding.

**Author’s response:** Thank you dear reviewer for your comment. We have added implications
